# Natural Variation in Clinical Isolates of Candida albicans Modulates Neutrophil Responses

**DOI:** 10.1128/mSphere.00501-20

**Published:** 2020-08-19

**Authors:** Madhu Shankar, Tricia L. Lo, Ana Traven

**Affiliations:** a Infection and Immunity Program, Biomedicine Discovery Institute, Monash University, Clayton (Melbourne), Victoria, Australia; b Department of Biochemistry and Molecular Biology, Biomedicine Discovery Institute, Monash University, Clayton (Melbourne), Victoria, Australia; University of Georgia

**Keywords:** *Candida albicans*, fungal infection, neutrophils

## Abstract

Neutropenia predisposes patients to life-threatening infection with Candida albicans, a commensal and opportunistic fungal pathogen. How phenotypic variation in C. albicans isolates dictates neutrophil responses is poorly understood. By using a panel of clinical C. albicans strains, here we report that the prototype strain SC5314 induces the most potent accumulation of reactive oxygen species (ROS) and neutrophil extracellular traps (NETs) by human neutrophils of all tested isolates. ROS and NET accumulation positively correlated with the degree of hyphal formation by the isolates, the hypha being the fungal morphotype that promotes pathogenesis. However, there was no correlation of ROS and NET accumulation with fungal killing by neutrophils. Fungal killing was also not correlated with phagocytosis levels or oxidative stress susceptibility of the isolates. The bloodstream isolate P94015 cannot make hyphae and was previously shown to be hyperfit in the murine gut commensalism model. Our results show that P94015 displays poor phagocytosis by neutrophils, the least ROS and NET accumulation of all tested isolates, and resistance to neutrophil-mediated killing. Our data suggest that reduced susceptibility to neutrophils is likely to be independent from a previously described genetic mutation in P94015 that promotes commensalism. Reduced clearance by neutrophils could benefit commensal fitness of C. albicans and could also have promoted the virulence of P94015 in the human patient in the absence of hyphal morphogenesis. Collectively, our study provides new insights into neutrophil interactions with C. albicans and suggests that studying diverse isolates informs knowledge of the relevant aspects of this key immune interaction.

**IMPORTANCE** Neutrophils are the key immune cell type for host defenses against infections with Candida albicans. C. albicans strains isolated from patients display large phenotypic diversity, but how this diversity impacts host-pathogen interactions with neutrophils is incompletely defined. Here, we show that important neutrophil responses, such as accumulation of reactive oxygen species and neutrophil extracellular traps, as well as the levels of phagocytosis and killing of the pathogen, differ when comparing diverse C. albicans isolates. A bloodstream patient isolate previously described as more suited to commensalism than pathogenesis in animal models is relatively “silent” to neutrophils and resistant to killing. Our findings illuminate the relationships between fungal morphogenesis, neutrophil responses, and C. albicans survival. Our findings suggest that host phenotypes of a commensally adapted strain could be driven by resistance to immune clearance and indicate that we should extend our studies beyond the “prototype” strain SC5314 for deeper understanding of *Candida*-neutrophil interactions.

## Abstract (precis)

Neutrophils are the key immune cell type for host defenses against infections with Candida albicans. C. albicans strains isolated from patients display large phenotypic diversity, but how this diversity impacts host-pathogen interactions with neutrophils is incompletely defined. Here, we show that important neutrophil responses, such as accumulation of reactive oxygen species and neutrophil extracellular traps, as well as the levels of phagocytosis and killing of the pathogen, differ when comparing diverse C. albicans isolates. A bloodstream patient isolate previously described as more suited to commensalism than pathogenesis in animal models is relatively “silent” to neutrophils and resistant to killing. Our findings illuminate the relationships between fungal morphogenesis, neutrophil responses, and C. albicans survival. Our findings suggest that host phenotypes of a commensally adapted strain could be driven by resistance to immune clearance and indicate that we should extend our studies beyond the “prototype” strain SC5314 for deeper understanding of *Candida*-neutrophil interactions.

## INTRODUCTION

Fungal infections are a global health care problem affecting countries around the world (1, 2). Candida albicans is one of the most common fungal pathogens and a recurrent member of the human microbiota (3). Increased load of C. albicans can cause oral and vaginal thrush, with more than 130 million women affected by recurrent *Candida* vaginitis with an estimated yearly economic loss that could reach around 14 billion U.S. dollars (4). C. albicans can additionally cause systemic invasive infections in hospitalized patients with mortality rates between 10 and 40% (2, 5). Immunosuppression, invasive treatments, and the use of indwelling devices are thought to be important reasons for increased frequency of serious *Candida* infections over the last few decades (5).

As an opportunistic pathogen, a key “opportunity” for C. albicans comes from low neutrophil counts (neutropenia), which leads to a risk of gut-derived invasive candidemia (6, 7). This condition is associated with cancer chemotherapy and emphasizes the importance of neutrophils in maintaining commensal levels of gut colonization and for defense against C. albicans (8–10). Upon increased fungal load or breach of defensive tissue barriers, neutrophils are recruited to infection sites where they kill C. albicans by a combination of mechanisms. These include phagocytosis, degranulation to release toxic mediators (such as neutrophil elastase, myeloperoxidase, and defensins), and production of reactive oxygen species (ROS) to kill the fungus by oxidative processes. In addition to these intracellular mechanisms, neutrophils also display extracellular candidacidal activity. They do so through the formation of neutrophil extracellular traps (NETs). NET formation entails extracellular release of neutrophil-derived chromatin structures that physically contain C. albicans and kill it through NET-associated antimicrobial factors, such as the metal chelator calprotectin (11, 12).

An important biological characteristic of C. albicans is growth in different cellular morphologies. These include yeast cells that divide by budding, filamentous forms resembling chains of elongated cells (named pseudohyphae), and true hyphal filaments (13–15). C. albicans switches dynamically between these forms depending on environmental attributes, including types and levels of nutrients, and the concentration of oxygen and carbon dioxide (14, 15). In the murine model of gut commensalism, both yeast and hyphae occupy the gut depending on the location that was imaged (16, 17), and analyses of mutants that are morphologically “locked” in one form suggest that both yeast and filaments play roles in commensalism and pathogenesis (14, 17–23). Having said that, genetic mutations that promote growth in yeast form promote colonization of the murine gut (24–26), while hyphae foster pathogenesis (14, 15, 20, 21), suggesting that hyphae are more “dangerous” than yeast cells to the host. This conclusion is also reflected in the way in which morphology dictates the interactions of C. albicans with the immune system and epithelial cells, whereby hyphae tend to be more potent inducers of host defense responses than yeast (27–30).

With respect to neutrophils, both hyphae and yeast cells of C. albicans are susceptible to killing and can trigger responses such as ROS and NET production (11). It has been shown that neutrophil ROS production and secretion of the cytokine interleukin-8 (IL-8) are controlled by the morphology of C. albicans in a manner depending on infection load: yeast cells induce more potent responses at low infection loads and hyphae do so at high infection loads (31). The encounter of neutrophils with hyphae triggers more robust NET release than that with yeast cells (11, 32). It has been proposed that this difference is due to the ability of neutrophils to phagocytose yeast cells, which suppresses the pathways needed for NET formation (32). Conversely, the inability of neutrophils to phagocytose large hyphae is thought to promote the formation of NETs (32). C. albicans resists neutrophil attack by utilizing stress pathways that enable survival of oxidative and nitrosative stress, detoxification of ROS by fungal superoxide dismutase (SOD) enzymes, and metabolic adaptation to the phagosome (33–35).

To date, most mechanistic studies of neutrophil responses to C. albicans have been performed using a prototype clinical isolate called SC5314. This approach ignores the fact that the diversity of C. albicans present in human populations is substantial. A recent genomics project analyzed a collection of clinical C. albicans isolates that included SC5314 and 20 other strains from various infection sites (25). These strains showed genomic differences and also displayed different phenotypes with respect to important cell biology, including resistance to various stressors and the ability to grow as true hyphae (25). This set of strains has been profiled for virulence in the murine bloodstream model, where they also showed considerable diversity (36). The potential impact of this phenotypic diversity on neutrophil interactions is unclear. Our study demonstrates considerable variability in neutrophil response to C. albicans strains and sheds light on several important aspects of this interaction.

## RESULTS

### Morphogenesis of C. albicans clinical isolates during coincubation with neutrophils.

**FIG 1:**
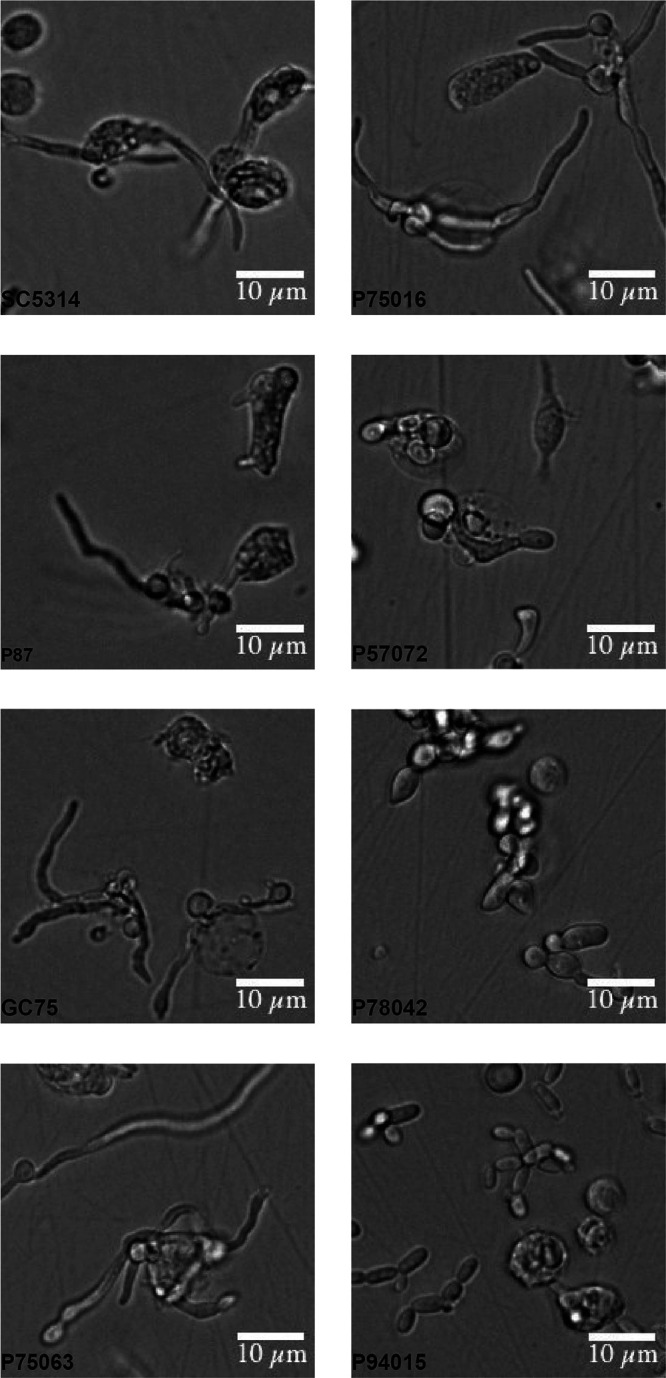
Cellular morphology of C. albicans isolates during interactions with neutrophils. Human neutrophils were challenged by the indicated C. albicans strains at an MOI of 1 for 6 h. Imaging was performed using an EVOS FL Auto microscope at ×40 magnification. Brightness and contrast were adjusted equally across all of the microscopy images using ImageJ software. Bar, 10 μm. Here, we show enlarged images of selected cells for each of the strains. The entire microscopy field is shown in Fig. S1.

To investigate how natural variation impacts C. albicans-neutrophil interactions, we selected eight clinical strains that belong to distinct clades, are from oral or bloodstream origin, and show a spectrum of cellular morphologies *in vitro* and diverse virulence profiles in the systemic murine infection model (25, 36). Our “control” was the prototype isolate SC5314, which belongs to clade I, forms robust hyphae, and is highly virulent in systemic mouse infections. Since C. albicans morphology was shown to impact neutrophil responses, we started by analyzing the cellular morphology of the isolates during neutrophil interactions. To that end, we challenged human neutrophils in RPMI medium for 6 h followed by imaging (note that the initial inoculum of C. albicans used to challenge neutrophils was in yeast form). Strains SC5314, P87, GC75, P75016, and P75063 formed hyphal filaments, P57072 and P78042 formed short pseudohypha-like cells, and P94015 remained in yeast-like morphology, displaying elongated cells and short chains of yeast cells (Fig. 1 and see also Fig. S1 in the supplemental material). The morphology of P94015 has been previously characterized as “intermediate/gray” and “opaque” yeast-like forms (24). The *in vitro* assessment of cell morphology of these isolates done by Hirakawa et al. (25) is broadly consistent with our analyses during coincubation with neutrophils.

10.1128/mSphere.00501-20.1 FIG S1 Images of C. albicans strains coincubated with neutrophils. Entire images of the coincubation experiments shown in Fig. 1. The black box indicates the part of the image that was enlarged to show in Fig. 1. Bar, 100 μm. Download FIG S1, TIF file, 1.9 MB. Copyright © 2020 Shankar et al. 2020 Shankar et al. This content is distributed under the terms of the Creative Commons Attribution 4.0 International license.

### Neutrophil ROS and NET production correlates with hyphal morphogenesis of the *Candida* isolates.

**FIG 2:**
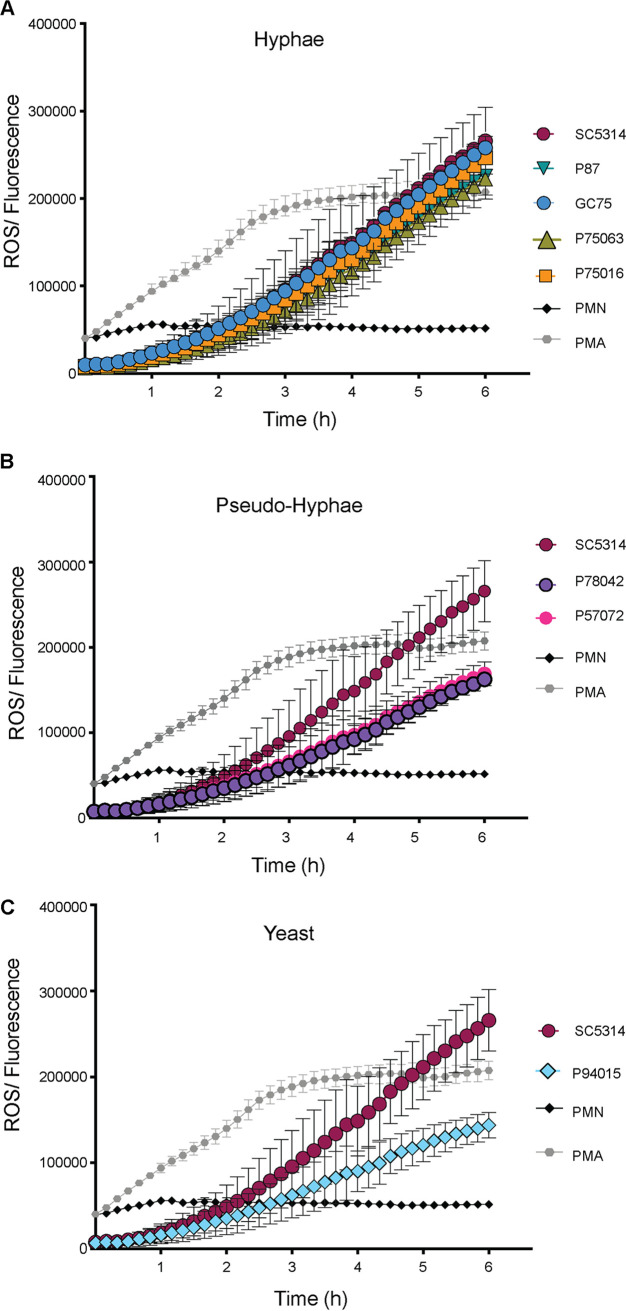
Induction of ROS production by neutrophils in response to the C. albicans isolates. Total ROS was detected over time for a period of 6 h, using H2DCFDA. All strains of C. albicans shown in the figure have been tested simultaneously, but the data are split into three graphs according to fungal morphology. Therefore, the data for the reference strain SC5314 and the positive and negative controls (100 nM phorbol 12-myristate 13-acetate [PMA] and neutrophils alone, polymorphonuclear cells [PMN]) are the same in the three graphs. The experiment was repeated with 3 independent donors. For each donor, 3 technical replicates were performed and the average for the technical repeats was calculated. Shown are the averages from the 3 donors and the SD. (A) C. albicans strains forming substantial hyphal cells are grouped together. (B) As in panel A but comparing SC5314 to strains that display pseudohyphal morphology. (C) As in panel A, but comparing SC5314 to P94015, which cannot form hyphae.

ROS are an important neutrophil weapon to counter pathogenic fungi (37). All C. albicans clinical isolates were able to induce ROS production by neutrophils, but the kinetics differed (Fig. 2). Strains SC5314, P87, GC75, P75016, and P75063 (which can make substantial hyphae) displayed similar kinetics of ROS production (Fig. 2A). These strains showed faster ROS accumulation and higher total ROS at the end of the experimental time course of 6 h than strains P78042 and P57072, which form short pseudohyphal filaments (Fig. 2B). Strain P94015 (yeast morphology) induced the smallest amount of ROS accumulation during neutrophil interactions (Fig. 2C).

**FIG 3:**
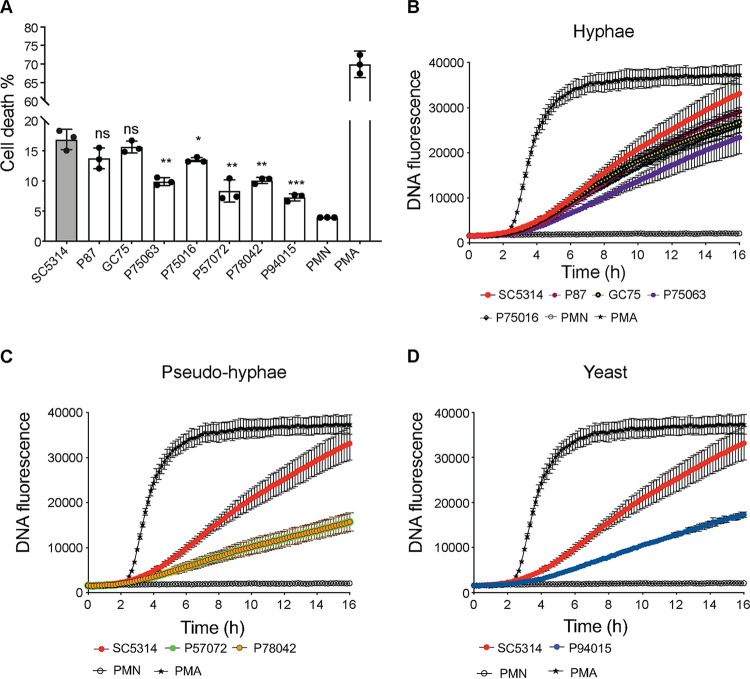
NET release in response to C. albicans isolates. (A) Percent death of neutrophils after 6 h of coincubation with the indicated strains of C. albicans (MOI of 1). The percent death was determined by considering neutrophils lysed with detergent (0.1% Triton X-100) as 100% death. The experiment was performed with 3 independent donors, in 3 technical replicates each (as in Fig. 2). Shown are the averages and standard error of the mean (SEM). The expressed *P* values are for the comparison with SC5314 (ordinary one-way ANOVA with Dunnett’s correction for multiple comparisons). *P* values are as follows: *, <0.05; **, <0.01; ***, <0.001; ns, not significant. (B) Infections of neutrophils were done as in panel A, and extracellular DNA release was monitored over time as described in Materials and Methods. Treatment with 100 nM PMA served as a positive control. All strains were assayed together, but the data are plotted in different graphs (B to D) accordingly to fungal morphology. The controls (PMA and neutrophils only [PMN]), as well as the data for the reference strain SC5314, are the same in all graphs. Shown in panel B are strains that form robust hyphae and filamentous morphologies. The experiment was performed with 3 different donors. Shown are averages and the SD. (C) As in panel B, but comparing SC5314 to strains that display pseudohyphal morphologies. (D) As in panel B, but comparing SC5314 to strain P94015, which displays yeast morphologies.

Next, we tested NET release as a further important antifungal mechanism (11). The process of NET release (so-called NETosis) causes cell death of neutrophils and can be thought of as a programmed cell death process (11). Therefore, we first determined the viability of neutrophils during coincubation with the various C. albicans isolates. As with ROS production, generally the hyphal isolates induced more neutrophil cell death, which was comparable to the death triggered by the prototype strain SC5314 (Fig. 3A). The yeast-morphology isolate P94015 induced the least death, while the pseudohyphal strains induced levels of neutrophil death that were in between those of SC5314 and P94015 (Fig. 3A). An exception was the hyphal strain P75063, which induced an amount of neutrophil cell death that was more comparable to the pseudohyphal strains than to other hyphal strains (Fig. 3A).

**FIG 4:**
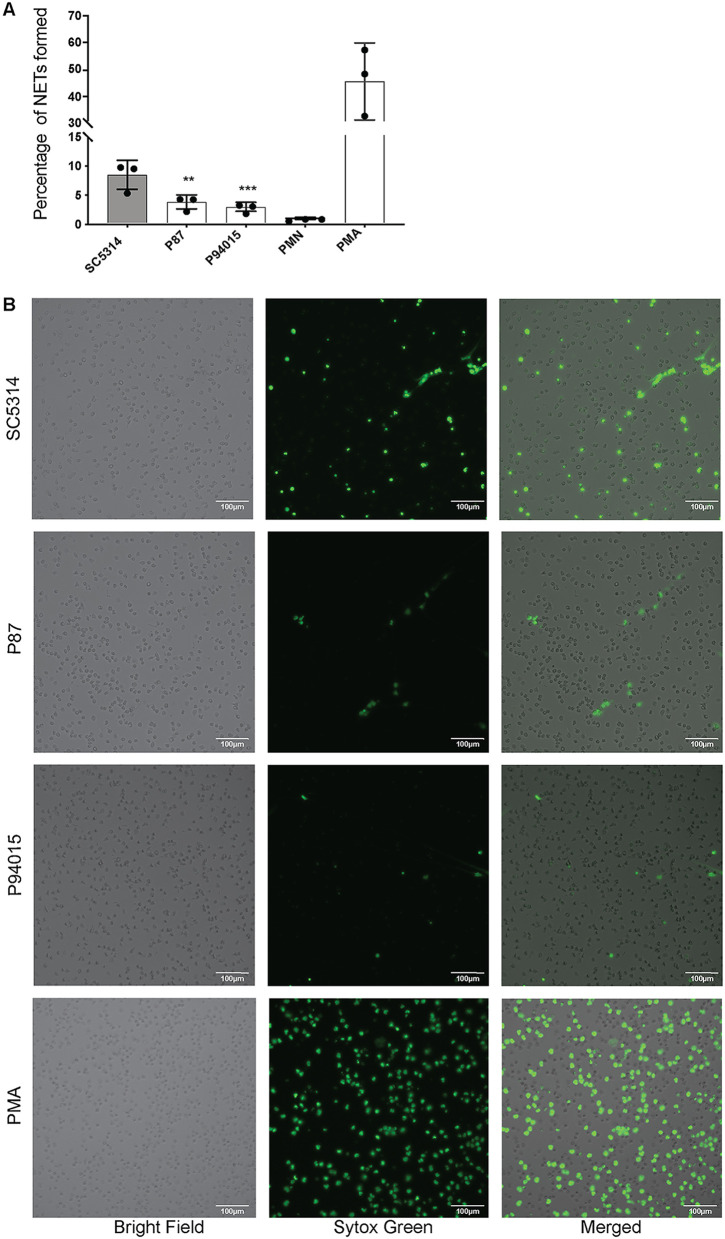
The C. albicans clinical isolates differ in their ability to trigger NET release. (A) Quantification of NETs released by neutrophils challenged with the indicated strains for 2 h (MOI of 1). Quantification was performed from microscopic images using ImageJ software. *n* = 3 donors, in 3 technical replicates each. Statistics analysis was performed using ordinary one-way ANOVA with Dunnett’s correction. The expressed *P* values are relative to the reference strain SC5314. *P* values are as follows: *, <0.05; **, <0.01; ***, <0.001. (B) Images of NET release following 2 h of coincubation of neutrophils with the indicated strains. NETs were visualized by staining with 160 nM Sytox Green. Bar, 100 μm.

Kinetic evaluation of extracellular DNA accumulation (which is a consequence of NET release) also showed a similar trend. The prototype strain SC5314 triggered the largest amount of extracellular DNA release followed by the other hyphal strains (Fig. 3B to D). The pseudohyphal isolates P78042 and P57072 induced lower levels than the hyphal strains, and the yeast-morphology strain P94015 induced the least extracellular DNA release (Fig. 3B to D). To further assess if the robustly hyphal strain P87 triggers fewer NETs than the prototype SC5314, we analyzed NET formation using fluorescence microscopy. These analyses showed that SC5314 triggered 5% more NET release than P87 (Fig. 4A and B) (note that the microscopy assay was performed 2 h after challenge because, at later time points, the NETs spread and overlap, making quantification difficult). Similarly, fluorescence microscopy showed that P94015 induced low NET formation (Fig. 4A and B).

**FIG 5:**
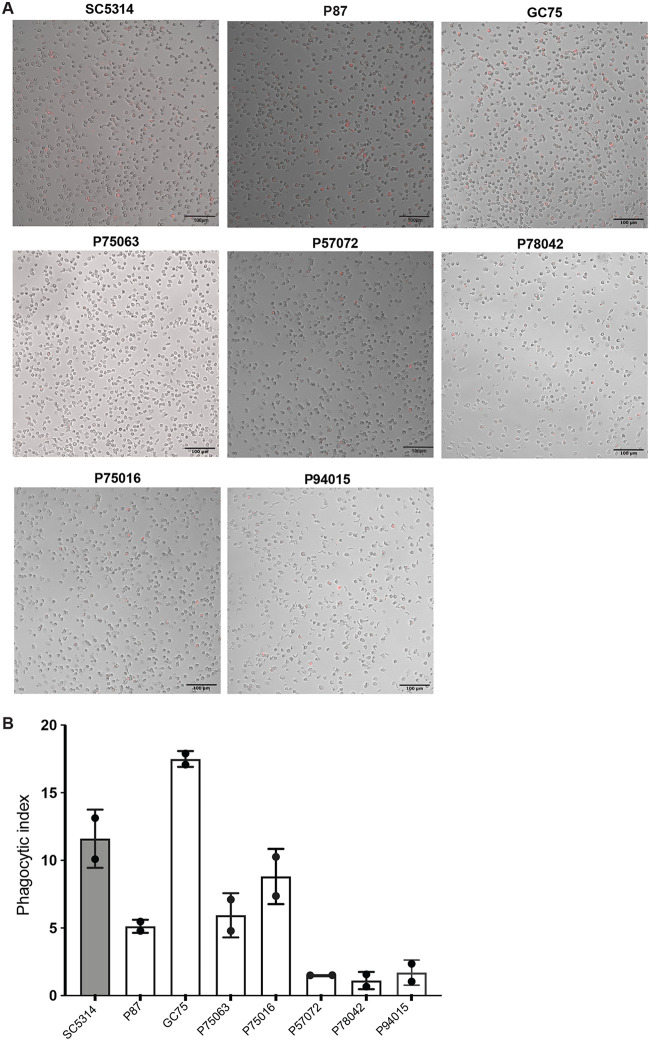
Phagocytosis of C. albicans isolates by neutrophils. (A) Microscopic images of phagocytosis were taken after 1 h of coincubation of C. albicans with neutrophils at ×20 magnification. C. albicans was stained with pHrodo red dye, which fluoresces only in the phagosome, but not if C. albicans is extracellular. Merged and enlarged images are shown here, and the individual bright-field and fluorescence images used to construct them are shown in Fig. S4. Bar, 10 μm. (B) Phagocytosis index was determined as described in Materials and Methods. The experiment was performed 2 independent times (each time, a different donor was used), and at least 100 neutrophils were counted in each experiment.

We next asked if the phagocytosis rates differed between the clinical strains. To assess phagocytosis, C. albicans was stained with pHrodo dye, which stains only phagocytosed cells (nonphagocytosed cells are not fluorescent) (Fig. 5 and Fig. S4). Most strains displayed lower phagocytosis rates than SC5314, with the exception of GC75, which had an average phagocytic index of 17, compared to 12 for SC5314 (Fig. 5B). Strains P57072, P78042, and P94015 were phagocytosed at the lowest levels of the strains we analyzed, with an average phagocytic index of 1.5, 1, and 1.67, respectively.

### Distinct susceptibility of the C. albicans isolates to killing by neutrophils.

**FIG 6:**
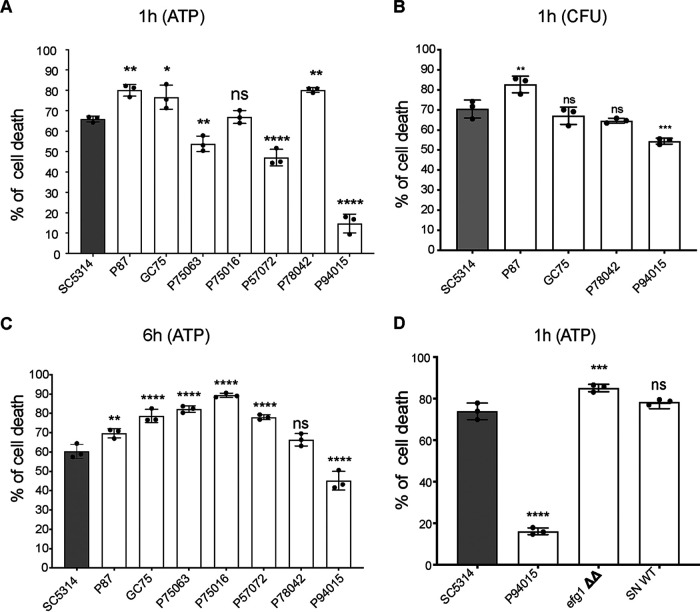
Distinct survival rates of C. albicans isolates in response to neutrophils. (A) Human neutrophils were challenged with C. albicans at an MOI of 1. C. albicans cell death was measured after 1 h of coincubation using the ATP assay. Percent death was determined relative to C. albicans grown without neutrophils for 1 h under the same growth conditions. (B) Experiment was performed as in panel A, but here the viability of C. albicans was determined by counting CFU. (C) Experiment performed as in panel A. Here, the coincubation of C. albicans with neutrophils was for 6 h, and the control was C. albicans grown alone for 6 h. (D) The experiment was performed as in panel B, using the ATP assay to determine the cell death of indicated C. albicans strains after 1 h of coincubation with neutrophils. Images comparing the cellular morphology of the *efg1*Δ/Δ mutant in the SN strain background compared to the clinical isolate P94015 are shown in Fig. S3. For all panels, *n* = 3 donors. The data points represent values for the 3 independent experiments (done in 3 technical repeats each). The error bar is the SEM. Statistical analysis was performed using ordinary one-way ANOVA, with Dunnett’s correction for multiple-comparison test. The expressed *P* values are relative to the reference strain SC5314. *P* values are as follows: **, <0.001; ***, <0.0001; ****, <0.00001; ns, not significant.

Phagocytosis and ROS and NET production all contribute to the killing of C. albicans by neutrophils. Therefore, we next sought to determine the susceptibility of the C. albicans isolates to killing by neutrophils. To that end, we challenged neutrophils at the multiplicity of infection (MOI) of 1 for 1 h, followed by differential lysing of the neutrophils and determination of C. albicans viability using the ATP assay (luminescent CellTiter-Glo kit) (Fig. 6A). To further confirm the ATP assay results, a subset of the strains representing each of the morphologies was also analyzed for viability after by determining CFUs (Fig. 6B; hyphae, SC5314, P87, and GC75; pseudohyphae, P78042; and yeast, P94015). Percent cell death was calculated relative to C. albicans grown without neutrophils for the same amount of time of 6 h. We also analyzed the 1-h data of neutrophils relative to the initial fungal inoculum, which showed that neutrophils were actually killing C. albicans in our assays and not simply inhibiting its growth (Fig. S2).

10.1128/mSphere.00501-20.2 FIG S2 Death of C. albicans strains in response to neutrophils. Neutrophils were challenged with C. albicans at an MOI of 1 for 1h. The viability of C. albicans was determined using the ATP assay. This is the same set of experiments as in Fig. 6A, but here the data for C. albicans death at 1 h is expressed as a percentage of the initial inoculum (0 h). Statistical analysis was performed using ordinary one-way ANOVA (correction using Dunnett’s multiple-comparison test). Shown are the *P* values relative to the prototype strain SC5314: **, <0.001; ***, <0.0001; ****, <0.00001; ns, not significant. Download FIG S2, TIF file, 0.1 MB. Copyright © 2020 Shankar et al. 2020 Shankar et al. This content is distributed under the terms of the Creative Commons Attribution 4.0 International license.

10.1128/mSphere.00501-20.3 FIG S3 Comparison of the cell morphology of the *efg1Δ/Δ* mutant and the P94015 strain. Cells were grown in in RPMI at 37°C, 5% CO_2_, for 3 h, and images were taken. Bar, 100 μm. Brightness and contrast were adjusted using ImageJ software, uniformly in all images. Download FIG S3, TIF file, 1.4 MB. Copyright © 2020 Shankar et al. 2020 Shankar et al. This content is distributed under the terms of the Creative Commons Attribution 4.0 International license.

10.1128/mSphere.00501-20.4 FIG S4 Phagocytosis of C. albicans by neutrophils. Bright-field and fluorescence images used to construct the merged images shown in Fig. 3. The black box indicates the part of the image that was enlarged to show in Fig. 5. Download FIG S4, TIF file, 1.5 MB. Copyright © 2020 Shankar et al. 2020 Shankar et al. This content is distributed under the terms of the Creative Commons Attribution 4.0 International license.

Relative to the prototype strain SC5314, the biggest difference in survival was for P94015 (yeast-like morphology), which showed significantly less death in both ATP and CFU assays and was overall the least susceptible to neutrophil killing of all the isolates that we tested (Fig. 6B). In the ATP assay, the average percent death of SC5314 was 65%, while for P94015 it was 15% (Fig. 6A). In CFU assays, SC5314 displayed 72% death, while P94015 displayed 55% death (Fig. 6B). Strains P75063 (hyphal morphology) and P57072 (pseudohyphal morphology) were also killed 10% and 15% less than SC5314, respectively, but more than P94015 (Fig. 6A). Strain P87 (hyphal morphology) was killed somewhat more than SC5314 in both ATP and CFU assays (80% versus 65% in the ATP assay and 82% versus 70% in the CFU assay) (Fig. 6A and B). Strains GC75 (hyphal morphology) and P78042 (pseudohyphal morphology) displayed higher killing than SC5314 in ATP assays (between 75% and 80% death) (Fig. 6A) but not when CFU were measured (Fig. 6B). Therefore, we considered them to be killed at an overall rate similar to SC5314.

We next tested the survival of the C. albicans isolates at 6 h postchallenge to address if distinct susceptibilities to neutrophils are maintained after a prolonged incubation and after NET release starts. The higher resistance of P94015 to neutrophils than of the other strains was also seen at the 6-h time point (Fig. 6C). P94015 showed between 15% and 45% more resistance to neutrophil killing than the other strains. The pseudohyphal strain P78042 was killed to a similar extent as SC5314 (Fig. 6C). All the other strains showed higher percentages of death than SC5314 at 6 h after challenge with neutrophils (Fig. 6C). Therefore, only P94015 is more resistant to neutrophils than SC5314 after prolonged incubation.

P94015 harbors a homozygous loss-of-function mutation in *EFG1*, the gene that encodes a C. albicans transcriptional activator required for hyphal morphogenesis under several (although not all) conditions (25). This isolate is especially fit for colonizing the gut in a commensalism model in mice (25), which is consistent with other studies showing that mutations that inhibit hyphal morphogenesis promote commensalism (16, 26). A homozygous deletion mutant of *EFG1* (*efg1Δ/Δ*) in the SN152 strain background (which is derived from SC5314) displayed morphology similar to P94015, in that cells remained in yeast-like form and formed some short chains of yeast cells (though fewer chains were observed than with P94015) (Fig. S3). However, unlike P94015, the *efg1Δ/Δ* mutant was not resistant to killing by neutrophils (Fig. 6D).

**FIG 7:**
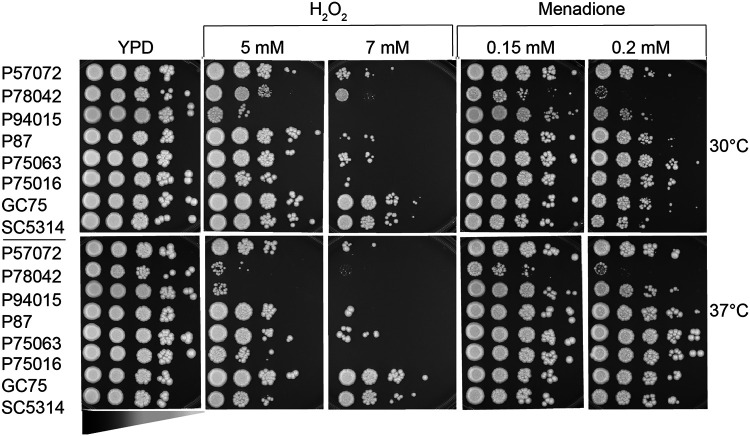
Oxidative stress susceptibility of the C. albicans clinical isolates. Fungal cultures of the indicated C. albicans strains were spotted in serial dilutions onto YPD plates containing hydrogen peroxide (H_2_O_2_) or menadione (0.15 mM and 0.2 mM). The C. albicans strains are spotted from higher to lower concentrations as depicted by the wedge. Growth was monitored at 30 or 37°C, and images were taken after 2 days. Shown are representative images from three independent experiments.

The ability of C. albicans to survive neutrophil attack depends on its ability to detoxify ROS and survive oxidative stress through activation of stress signaling pathways (33). The clinical strains used here have been reported to have distinct hydrogen peroxide susceptibility (25), but we wanted to test this more comprehensively, particularly with respect to growth at host temperature of 37°C. Of the strains that we tested, P94015 was the most susceptible to hydrogen peroxide, followed by P78042 (Fig. 7). P94015 was also marginally susceptible to menadione at 30°C, while P78042 was strongly susceptible to menadione (Fig. 7). Several of the other strains were also more susceptible to hydrogen peroxide than was the prototype strain SC5314, particularly at the higher dose of 7 mM H_2_O_2_ and during growth at host temperature of 37°C (Fig. 7). The exception was GC75, which displayed hydrogen peroxide susceptibility that was comparable to SC5314 (Fig. 7).

### Production of TNF-α cytokine by neutrophils.

In addition to directly killing microbial pathogens, neutrophils make cytokines. Tumor necrosis factor alpha (TNF-α) is produced by neutrophils in response to C. albicans (38, 39). Therefore, a selection of the C. albicans isolates representative of the three morphologies was tested for their ability to induce TNF-α production by neutrophils (hyphae, SC5314, P87, and GC75; pseudohyphae, P57072 and P78042; and yeast, P94015). The prototype strain SC5314 was by far the most potent inducer of TNF-α (Fig. S5). Again, the yeast-morphology strain P94015 triggered the least pronounced response, with almost 10-fold-lower levels of TNF-α than those induced by SC5314 (Fig. S5). The other strains, namely, GC75, P87, P57072, and P78042, all induced lower TNF-α levels than SC5314, and no correlation between cell morphology and TNF-α release was observed.

10.1128/mSphere.00501-20.5 FIG S5 TNF-α production by neutrophils when challenged by different strains of C. albicans. TNF-α was detected using ELISA. The assay was performed twice independently in three technical replicates each; *n* = 2 are displayed as average and SD. Download FIG S5, TIF file, 0.1 MB. Copyright © 2020 Shankar et al. 2020 Shankar et al. This content is distributed under the terms of the Creative Commons Attribution 4.0 International license.

## DISCUSSION

In this study, we assessed the impact of natural variation and phenotypic divergence in C. albicans clinical isolates on the interaction with neutrophils, a key immune cell type responsible for controlling *Candida*. The clinical isolates that we studied vary greatly in several phenotypes, including virulence in the mouse systemic model, stress resistance, and hyphal morphogenesis (25, 36) (Fig. 1). Here, we show for the first time that this panel of isolates also differs in their interactions with neutrophils. Specifically, they show distinct levels of phagocytosis, accumulation of ROS and NETs, survival upon neutrophil attack, and production of TNF-α cytokine. Overall, strain SC5314 induced the most pronounced responses from neutrophils for the pathways that we tested. This shows that SC5314, while considered a prototype C. albicans strain, is on the extreme high end for neutrophil responses compared to other isolates (although not an outlier). This should be kept in mind when generalizing conclusions.

Induction of ROS and NETs by neutrophils was positively correlated with the degree of hyphal formation by the clinical isolates in our study. While both ROS-dependent and ROS-independent mechanisms of NET release have been described (40, 41), our data showing that the C. albicans isolates inducing higher ROS also induced more NET release and vice versa support a link between NET and ROS production. Although the clinical isolates are not isogenic and therefore features other than morphogenesis could have an impact on their ability to trigger ROS and NETs, previous work comparing isogenic strains is consistent with our conclusion that hyphae lead to more ROS and more robust NET responses (32, 39). However, we found no correlation between the levels of ROS, NETs, phagocytosis, and *Candida* hyphal morphogenesis and the extent of killing of the isolates by the neutrophils. For example, challenging neutrophils with isolate P78042 led to less phagocytosis and less ROS and NET accumulation than challenge with SC5314, but this isolate was not killed less than SC5314. P57072 was phagocytosed less than SC5314 and induced less ROS and NETs; although it was less susceptible to neutrophils at 1 h, it was killed 20% more than SC5314 if the coincubation was extended to 6 h. Also, P87 was phagocytosed less than SC5314 and induced similar levels of ROS and somewhat fewer NETs but was more susceptible to killing at both 1 and 6 h of coincubation. Survival of neutrophil attack was also not correlated with *in vitro* oxidative stress susceptibility of the isolates. For example, P94015 was the least susceptible to neutrophils but the most susceptible to oxidative stress. P78042 was more susceptible to oxidative stress than SC5314 but was not killed more by neutrophils. Collectively, our results show that, while the counter of neutrophils with C. albicans hyphae promotes NET and ROS accumulation, this does not necessarily translate to more effective killing of the fungal pathogen. Clearly, multifactorial mechanisms dictate the outcome of the neutrophil-C. albicans interactions (11, 25, 32, 35, 40). It is likely that the distinct pressures that these clinical isolates faced in their respective patient host niches determined their adaptations to neutrophil interactions in a complex manner.

Our data inform the relationship between phagocytosis of C. albicans by neutrophils and NET formation (41). By using SC5314 and a yeast-locked mutant derived from it, it has been shown that the process of phagocytosis inhibits the process of NETosis (32). This result offers an explanation for why yeast cells do not trigger NETosis (they are small and efficiently phagocytosed), while hyphae do (they are big and cannot be phagocytosed) (32). By using the C. albicans hypha-deficient mutant *hgc1Δ/Δ* strain in experiments in which a modified transwell system blocked phagocytosis while physical interaction between yeast and neutrophils could occur, this study showed that yeast-morphology cells can trigger NETosis if their phagocytosis is prevented by a physical barrier (32). This result further supports the authors’ conclusion that if phagocytosis of C. albicans is poor, then NETosis ensues, and also suggests that size, rather than other difference between yeast and hyphal morphologies, is the main factor determining NETosis (32). This result would also suggest that NETosis is not specifically triggered by the “frustrated phagocytosis” of hyphae (i.e., the membrane processes that occur when neutrophils try to engulf hyphae but do not succeed), since “frustrated phagocytosis” would not occur in the aforementioned transwell experiments with neutrophils and yeast cells. Our results question the conclusion that poor phagocytosis is a predictor of robust NETosis. The C. albicans clinical isolate P94015 cannot form hyphae and remains in yeast-like morphology and is poorly phagocytosed by neutrophils, but nevertheless, it does not trigger robust NETosis. Similarly, strains P57072 and P78042 (which form short pseudohyphae) are also poorly phagocytosed by neutrophils but do not trigger robust NETosis. In contrast, the clinical isolates that can form substantial hyphae triggered more NETosis in our experiments, showing that hyphal attributes additional to size and lack of phagocytosis are needed to drive NETosis.

Our data also provide some insights into the behavior of strain P94015. Although the provenance of P94015 is from human bloodstream, this strain is avirulent following bloodstream infections in mice (36), yet well adapted to commensal colonization in the mouse gut model (25). It has been speculated that the absence of a competent immune system in the patient might have enabled an otherwise avirulent strain that lacks hyphae, such as P94015, to reach the bloodstream (25). We now show that P94015 is relatively more “silent” to human neutrophils than the other strains we tested, as it was the least phagocytosed strain and it induced the fewest ROS and NETs. Importantly, P94015 was also the most resistant to neutrophil-mediated killing of the strains that we tested. These phenotypes could have contributed to its survival in the human patient.

Why is strain P94015 resistant to neutrophils? Accumulation of ROS in response to P94015 was reduced compared to SC5314 and the other strains, but *in vitro* P94015 was among the most susceptible to oxidative stress of the strains that we analyzed. This makes it difficult to conclude that differences in oxidative killing are responsible for the observed relative resistance of P94015 to neutrophils. Instead, we propose that the lower phagocytosis of P94015 (which would reduce intracellular killing) and lower induction of NETs (which would reduce extracellular killing) are collectively contributing to its increased survival. It is also likely that additional mechanisms contribute to neutrophil resistance of P94015. We conclude this because P78042, a strain that showed phenotypes very similar to P94015 (i.e., less phagocytosis, less NET and ROS induction, and relatively high susceptibility to oxidative stress *in vitro*), did not display lower killing by neutrophils than SC5314. We further show that the resistance to neutrophils of P94015 is unlikely to be driven by its loss-of-function mutation in the transcription factor *EFG1*, which has been suggested to be the reason for its commensal adaptation (25). This is because, unlike P94015, the *efg1* mutant in the SN lab strain background (derived from SC5314) was not more resistant to neutrophil killing than its wild-type control in our experiments. However, it still remains possible that *EFG1* plays a role in neutrophil resistance in the genetic background of P94015 but not SC5314. The idea that resistance to clearance by neutrophils might promote commensalism is in line with the fact that commensally fit mutants of C. albicans have been shown to trigger less death of mouse macrophages and human gut epithelial cell lines (26) and the proposition that reduction in hypha-specific adhesion and lytic enzyme expression contributes to commensalism by reducing immune clearance (16). At present, we do not know if P94015 is well suited for gut colonization in humans, because all studies with this strain with respect to commensalism come from mice. We have done all of our experiments with human neutrophils. There are differences in the interactions of human versus murine neutrophils with C. albicans (42), and we do not know how P94015 behaves with mouse neutrophils. Keeping these disclaimers in mind, our data with P94015 support the idea that reduced susceptibility to neutrophils might be one of the factors contributing to commensal fitness of C. albicans in the mammalian host.

## MATERIALS AND METHODS

### Isolation of human neutrophils.

Blood was collected from healthy volunteers. Neutrophil isolation was performed using a previously described method (43). Briefly, blood was collected on a Li-heparin 6-ml Vacutainer and neutrophils were isolated using negative selection with the EasySep direct human neutrophil isolation kit (Stemcell Technologies, Cambridge, United Kingdom). Once isolated, human neutrophils were suspended in RPMI 1640 without phenol red containing 1% human serum, which was also the medium in which all assays were performed. Before every experiment, neutrophils were counted and evaluated for viability using trypan blue staining and diluted to the desired concentration.

### Infecting neutrophils with C. albicans.

The C. albicans isolates used in this study are described in the work of Hirakawa et al. (25). They were obtained from BEI Resources, NIAID, NIH. For the neutrophil challenge experiments, the cultures of C. albicans were grown by patching single colonies on a YPD plate (1% yeast extract, 2% peptone, 2% glucose, 2% agar, 80 μg/ml uridine), followed by incubation at 30°C overnight for 12 h. Cells were taken from the plates and resuspended in phosphate-buffered saline (PBS), counted in a hemocytometer, and then used for assays with neutrophils at an MOI of 1. The inoculum of C. albicans used for challenging neutrophils was in yeast morphology.

### ROS measurements.

ROS was measured by using the chemically reduced form of fluorescein, 2′,7′-dichlorodihydrofluorescein diacetate (H2DCFDA), as indicator for ROS (ThermoFisher Scientific). Neutrophils were stained with H2DCFDA for 10 min in the dark and then washed twice using 1× PBS. The neutrophils were seeded into a black 96-well plate and infected with C. albicans at an MOI of 1. For a positive control, phorbol 3-myristate (PMA) was added to neutrophils at 100 nM. The assay plate was incubated at 37°C and 5% CO_2_, in a Tecan SparkM plate reader. Fluorescence readings were taken every 10 min for 16 h at excitation/emission (Ex/Em) of ∼492 to 495/517 to 527 nm, respectively.

### *Candida* cell death.

Cell viability of the C. albicans isolates in the presence of human neutrophils was assessed using ATP as the measure of viability or by counting CFU. Cell viability/cell death was measured using the CellTiter-Glo kit (Promega), according to the manufacturer’s instructions. Briefly, neutrophils challenged with C. albicans were added to black-bottom or black clear-bottom 96-well plates. Plates were incubated at 37°C and 5% CO_2_ for 1 h or 6 h based on the assay requirements. After the incubation, 0.1% Triton X-100–cold H_2_O was added to all wells, followed by vigorous pipetting in order to lyse the neutrophils. Plates were incubated for 10 min. The plates were washed twice with 1× PBS. Then, 50 μl of RPMI 1640 was added to the plates, followed by addition of equal volume of CellTiter-Glo reagent. Plates were incubated in the dark with slight shaking for 20 min. The plates were analyzed for ATP reads in the Tecan SparkM plate reader. Viability was recorded as a measure of luminescence. In order to calculate viability, the luminescence of lysed neutrophils was subtracted from values of *Candida* coincubated with neutrophils. Percent death was calculated by 100 − [(luminescence of *Candida* in coculture/luminescence of *Candida* in control group) × 100].

Candidacidal activity of neutrophils was also measured by counting survivors using CFU. After coculture, a 100-μl aliquot was taken out into fresh tubes. Neutrophils were lysed by adding cold sterile water (500 μl) and incubating for 10 min. Remaining C. albicans cells were then serially diluted, and using glass beads, 100 μl of cell suspension was spread on YPD plates and counted after 48 h of incubation at 30°C. The percent killing of *Candida* was calculated using the formula [100 − (CFU of *Candid*a in coculture/CFU of *Candida* in control group)] × 100. Data generated from three technical repeats were averaged to represent one value per donor. Neutrophils from three independent donors were used for each assay.

### DNA fluorescence assay.

Extracellular DNA was measured as a proxy for quantifying NET formation, using methods previously described (38). In brief, 1 × 10^5^ cells were seeded in a black-bottom 96-well plate. DNA binding stain Sytox Green (2.5 μM; ThermoFisher Scientific, USA) was added to the plate. C. albicans strains were added to plates with neutrophils at an MOI of 1. PMA treatment (100 nM) was used as control. In a separate well, as control for 100% lysis, neutrophils were lysed with Triton X-100 (0.1%). Fluorescence was measured every 10 min in the Tecan SparkM microtiter plate read for 16 h and calculated as percentage of the 100% lysis control.

### Phagocytosis assay.

*Candida* cells were stained with pHrodo red succinimidyl ester (0.2 mM) for 10 min in the dark. The cells were washed three times in 1× PBS and then counted using a hemocytometer. Human neutrophils were added to the 96-well plate at a concentration of 1 × 10^6^ cells/ml and incubated at 37°C for 30 min to allow cells to attach to the plate. Stained *Candida* cells were added to the plates at an MOI of 1. The plate was incubated at 37°C and 5% CO_2_ for 1h in the dark. Microscopic images were taken at 20× on a Leica live-cell DMi8 microscope. ImageJ software was used to count the bright field (neutrophils) and red channel (phagocytosed *Candida*). Phagocytic index was calculated using the formula (total number of engulfed cells/total number of counted neutrophils) × (number of neutrophil-containing engulfed cells/total number of counted neutrophils) × 100.

### Microscopy image analysis.

C. albicans strains and human neutrophils were coincubated in RPMI 1640 without phenol red containing 1% human serum. Sytox Green, a DNA staining dye that does not cross an intact cellular membrane, was added to the well of the plate (160 nM) just before imaging. An image of each sample was taken using a Leica live-cell DMi8 microscope at 20× magnification. Five random images from each well were taken. The ImageJ software was used to count NETs formed and total neutrophils per field of view. The percent NETs formed was calculated by dividing the number of NETs formed by total number of neutrophils.

### Ethics statement.

The isolation of neutrophils from human blood was in accordance with MUHREC (Monash University Human Research Ethics Committee) project numbers 9572 and 21685.

### Statistical analysis.

Statistical analysis was conducted using GraphPad Prism 8 software, and *P* values less than 0.5 were considered significant. For assays involving neutrophils from 3 donors, the technical replicates for each donor were averaged to one data point and data from three donors were analyzed with standard deviation (SD). Comparisons of different clinical strains with lab strain SC5314 were performed using one-way analysis of variance (ANOVA) multiple-comparison analysis as specified in the figure legends. In all comparisons, sample size is specified in the figure legends and a *P* value of <0.05 was considered significant (*, *P* < 0.05; **, *P* < 0.01; ***, *P* < 0.001; ****, *P* < 0.0001; ns, not significantly different).
