# Supplementary figures and images for: Natural Variation in Clinical Isolates of Candida albicans Modulates Neutrophil Responses
Source: mSphere. 2020 Aug 19;5(4):e00501-20. doi: 10.1128/mSphere.00501-20 (PMC7440844; doi:10.1128/mSphere.00501-20)

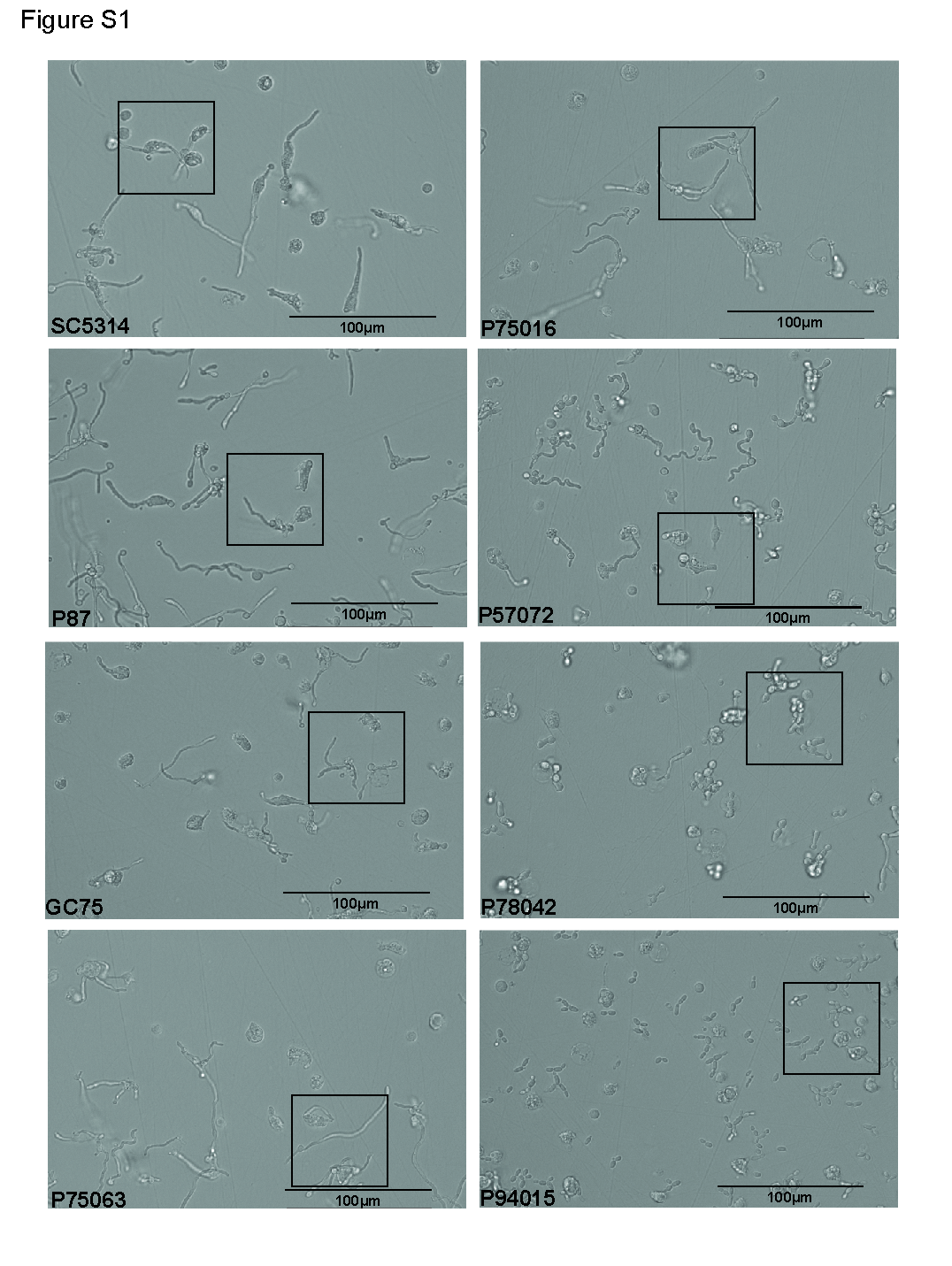

Supplement: FIG S1 [file mSphere.00501-20-sf001.tif]

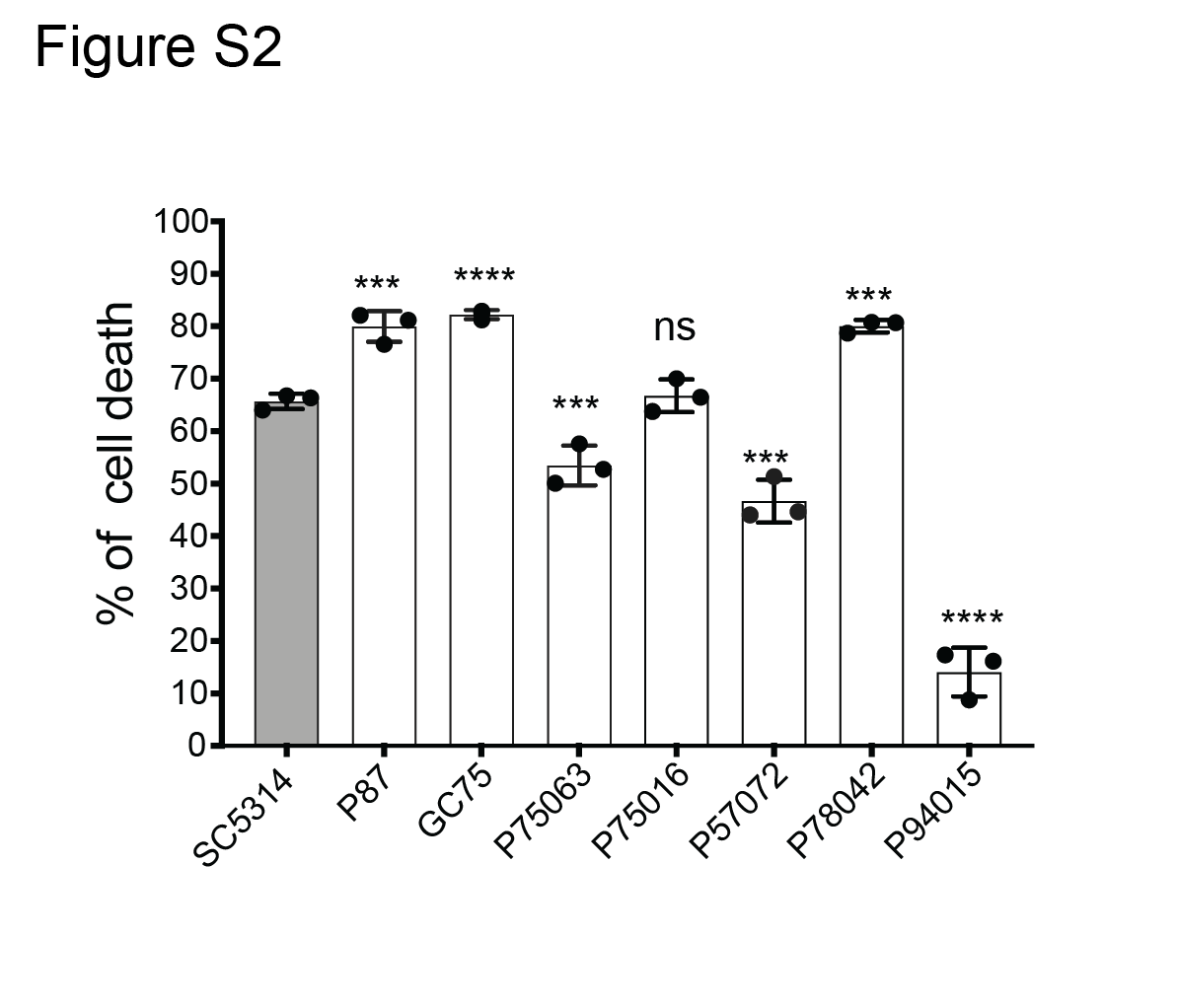

Supplement: FIG S2 [file mSphere.00501-20-sf002.tif]

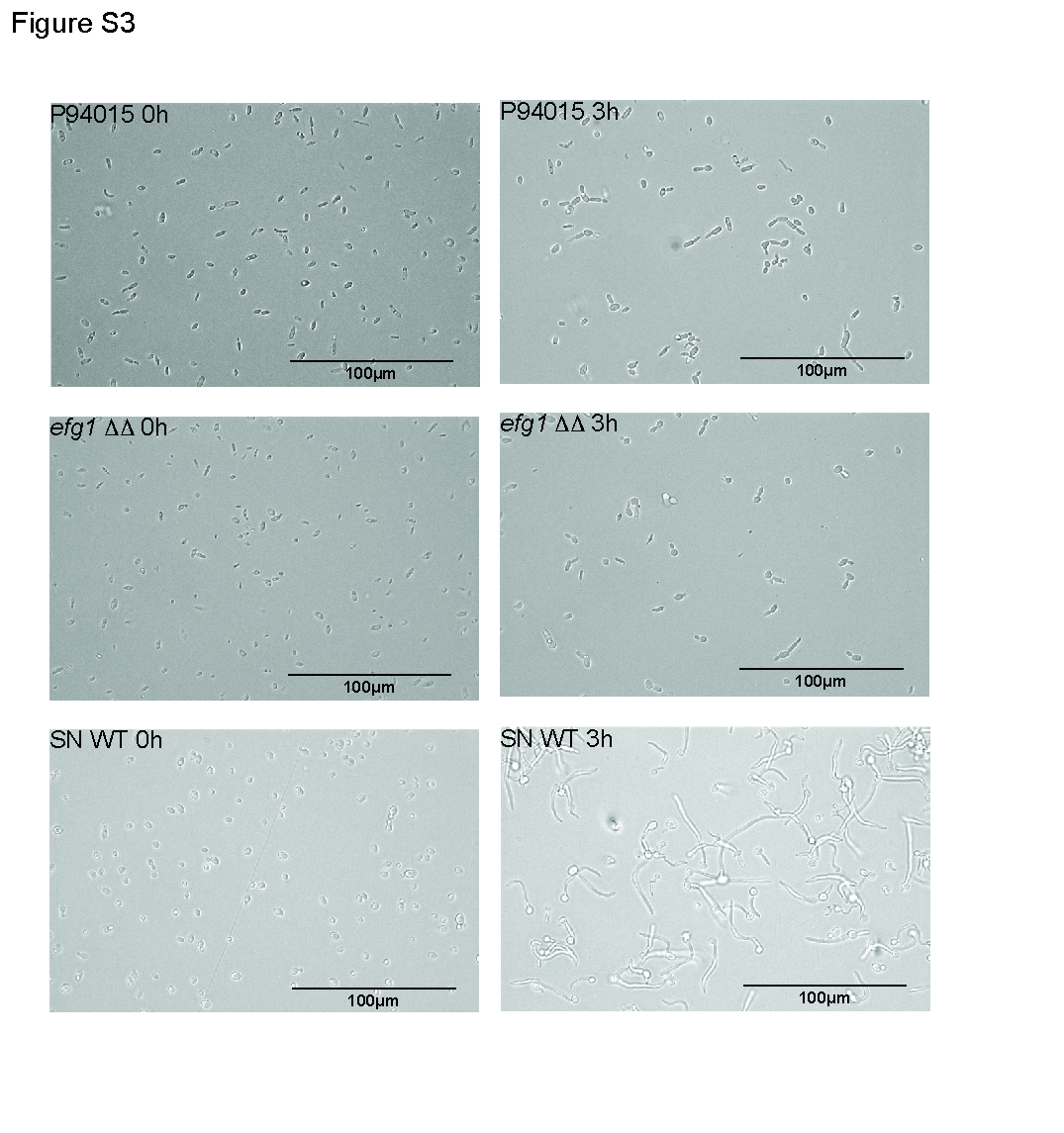

Supplement: FIG S3 [file mSphere.00501-20-sf003.tif]

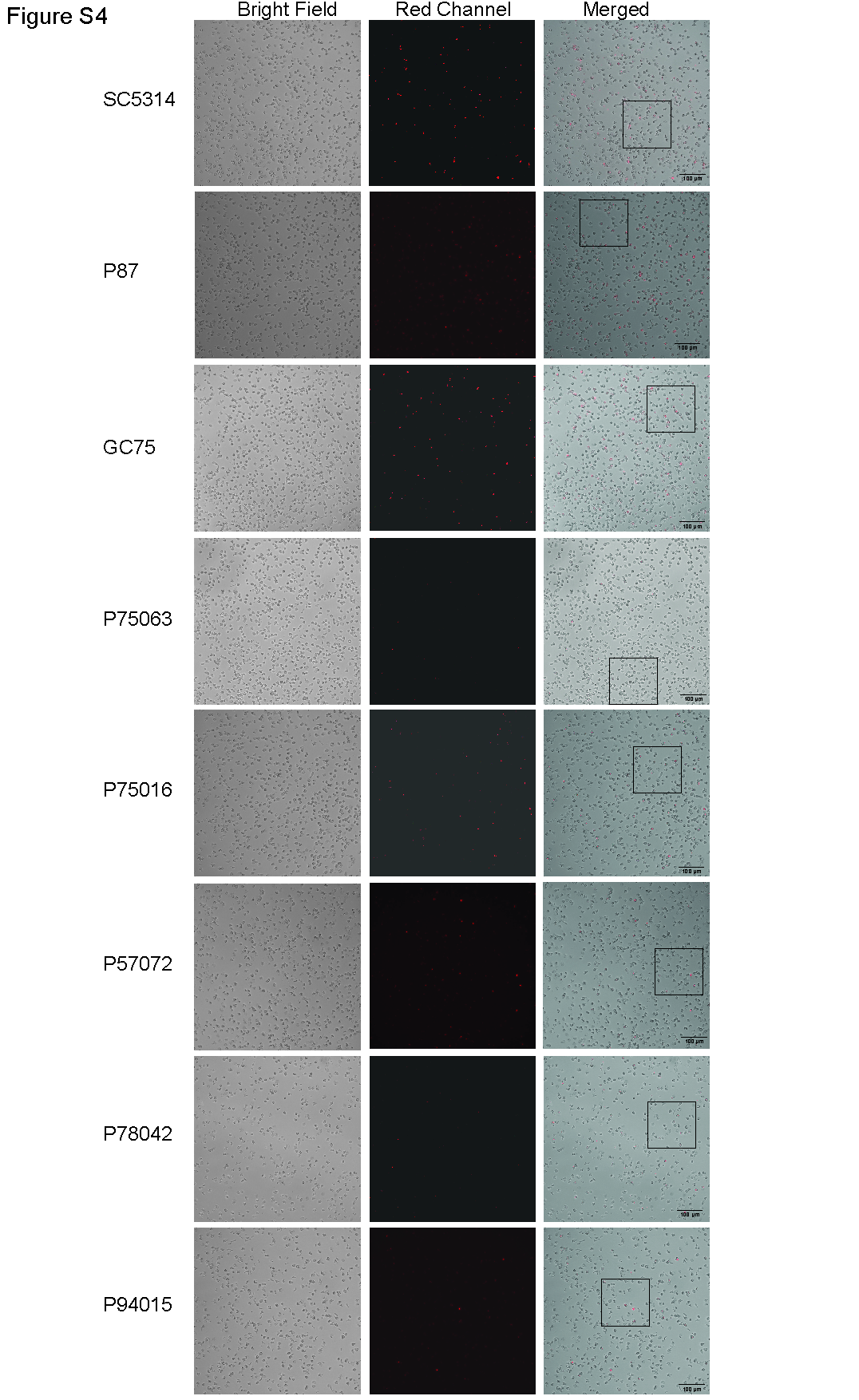

Supplement: FIG S4 [file mSphere.00501-20-sf004.tif]

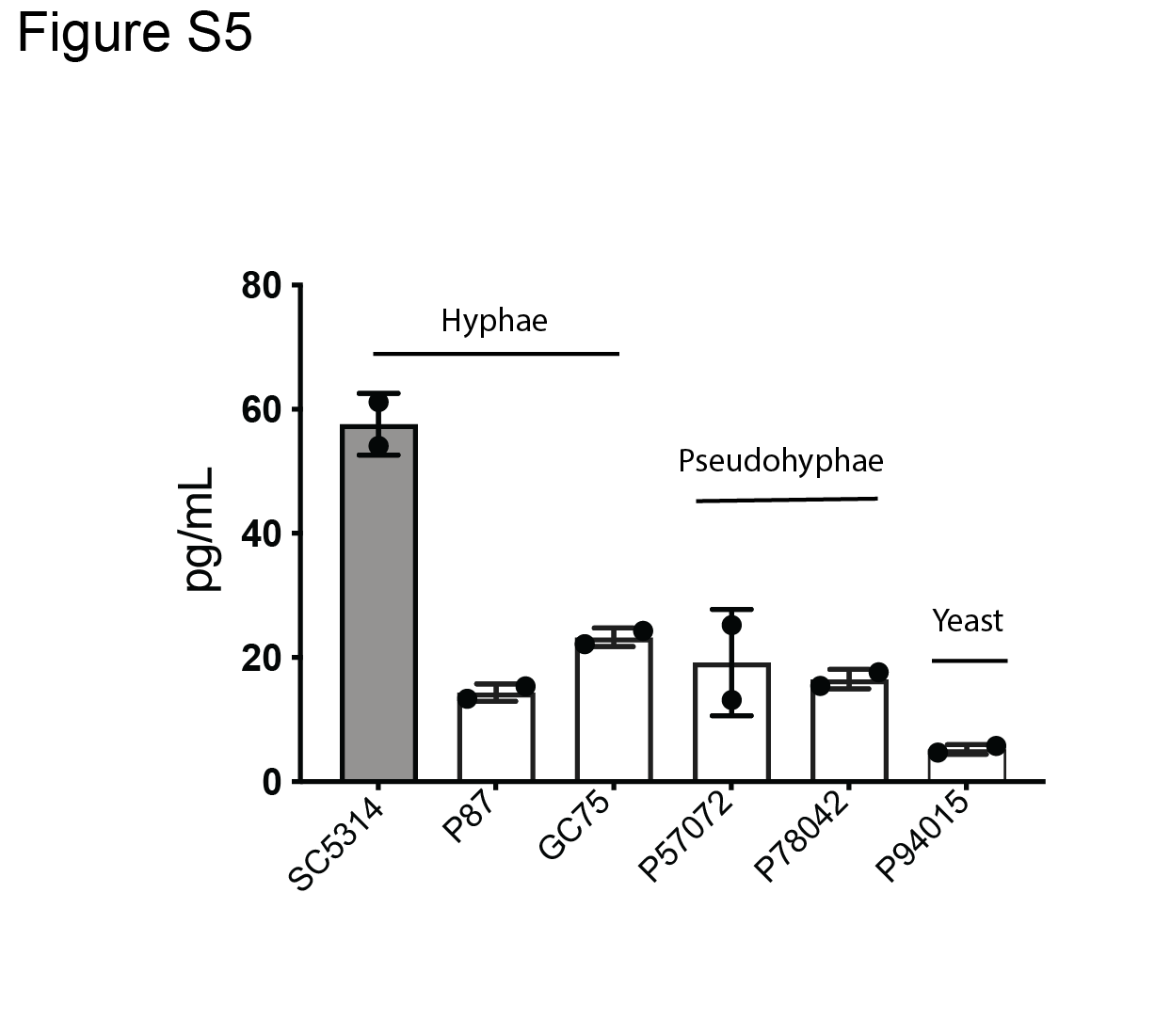

Supplement: FIG S5 [file mSphere.00501-20-sf005.tif]
